# DYT-*PRKRA* Mutation P222L Enhances PACT’s Stimulatory Activity on Type I Interferon Induction

**DOI:** 10.3390/biom12050713

**Published:** 2022-05-17

**Authors:** Lauren S. Vaughn, Kenneth Frederick, Samuel B. Burnett, Nutan Sharma, D. Cristopher Bragg, Sarah Camargos, Francisco Cardoso, Rekha C. Patel

**Affiliations:** 1 Department of Biological Sciences, University of South Carolina, 700 Sumter Street, Columbia, SC 29208, USA; vaughnls@email.sc.edu (L.S.V.); frederk@email.sc.edu (K.F.); burnettsamuelb@gmail.com (S.B.B.); 2 Department of Neurology, Massachusetts General Hospital, Charlestown, MA 02129, USA; nsharma@partners.org (N.S.); bragg@helix.mgh.harvard.edu (D.C.B.); 3 Department of Internal Medicine, Federal University of Minas Gerais, Belo Horizonte 31270-901, Brazil; sarahcamargos@hotmail.com (S.C.); fecardosoc@gmail.com (F.C.)

**Keywords:** dystonia, DYT16, DYT-*PRKRA*, PACT, *PRKRA*, PKR, interferon, RIG-I

## Abstract

DYT-*PRKRA* (dystonia 16 or DYT-*PRKRA*) is caused by mutations in the *PRKRA* gene that encodes PACT, the protein activator of interferon (IFN)-induced double-stranded (ds) RNA-activated protein kinase (PKR). PACT participates in several cellular pathways, of which its role as a PKR activator protein during integrated stress response (ISR) is the best characterized. Previously, we have established that the DYT-*PRKRA* mutations cause enhanced activation of PKR during ISR to sensitize DYT-*PRKRA* cells to apoptosis. In this study, we evaluate if the most prevalent substitution mutation reported in DYT-*PRKRA* patients alters PACT’s functional role in induction of type I IFNs via the retinoic acid-inducible gene I (RIG-I) signaling. Our results indicate that the P222L mutation augments PACT’s ability to induce IFN β in response to dsRNA and the basal expression of IFN β and IFN-stimulated genes (ISGs) is higher in DYT-*PRKRA* patient cells compared to cells from the unaffected controls. Additionally, IFN β and ISGs are also induced at higher levels in DYT-*PRKRA* cells in response to dsRNA. These results offer a new avenue for investigations directed towards understanding the underlying molecular pathomechanisms in DYT-*PRKRA*.

## 1. Introduction

Dystonia is a heterogeneous group of movement disorders which causes repetitive and often painful movements of the affected limbs leading to compromised posture and gait patterns [1,2]. Mutations in the *PRKRA* gene cause one form of early-onset, progressive generalized dystonia (DYT-*PRKRA*) and some of the patients also present with parkinsonism (OMIM: DYT-*PRKRA*, 612067). Ten different mutations have been identified in *PRKRA* that cause DYT-*PRKRA* [3,4,5,6,7,8,9,10,11], which is characterized by progressive limb, laryngeal, and oromandibular dystonia accompanied in some by parkinsonism. Recently, bilateral striatal degeneration was reported in several DYT-*PRKRA* patients [10,11] and movement as well as neurodevelopmental abnormalities have been noted in mice carrying the *PRKRA* mutation [12]. Although DYT-*PRKRA* was originally described to have an autosomal recessive inheritance pattern [3], some dominantly inherited variants of *PRKRA* have also been reported [4,7].

The *PRKRA* gene encodes for PACT protein, which is expressed ubiquitously in all examined cell types at varying levels [13]. One of the well-characterized cellular functions of PACT is to activate the interferon (IFN) induced double-stranded RNA (dsRNA)-activated protein kinase (PKR) in response to cellular stress. PKR is a ubiquitous kinase [14,15] active under cellular stress conditions such as viral infection, oxidative stress, endoplasmic reticulum (ER) stress, inflammation, and serum deprivation [16,17]. PKR’s kinase activity stays latent until it is bound to one of its activators, either dsRNA during viral infections or PACT during non-viral cellular stress signals and once active, it phosphorylates the α subunit of the protein synthesis initiation factor 2 (eIF2α) to activate the integrated stress response (ISR) pathway [15,18,19]. ISR is an evolutionarily conserved pathway activated in eukaryotes by many different types of stress stimuli in order to restore cellular homeostasis [20]. While transient eIF2α phosphorylation is favorable for cellular survival, prolonged eIF2α phosphorylation is pro-apoptotic due to the upregulation as well as preferential translation of pro-apoptotic transcripts [21]. Thus, although ISR is primarily a pro-survival response to restore cellular homeostasis, exposure to severe stress drives signaling towards cellular death. Our previous studies have demonstrated that seven of the inherited DYT-*PRKRA* mutations increase cell susceptibility to ER stress through the dysregulation of the eIF2α stress response [22,23,24]. In accordance with our findings, subsequent reports also identified dysregulation of eIF2α signaling in DYT1, DYT6, and DYT11 dystonia [25,26,27,28], as well as in sporadic cervical dystonia [28], traumatic brain and spinal-cord injury-induced dystonia [29]. Dystonia caused by mutations in genes related to calcium physiology also have evidence of altered ER stress response [30].

Other than the PKR-regulated cellular stress response pathway, other pathways in which PACT is known to participate are the RNA interference (RNAi) pathway [31,32,33] and type I IFN induction via cytoplasmic pattern recognition receptors (PRR) that recognize, bind to, and are activated by dsRNA [34,35,36]. PACT’s functional role in the type I IFN induction in response to dsRNA is the focus of this study. The ability of cells to discriminate between self, nonpathogenic and non-self, pathogenic molecules is central for an effective innate immune system [37]. Detection of pathogenic molecules is performed by PRRs, which specialize in detection of pathogen-associated molecular patterns (PAMPs) [38,39]. After detection of pathogen associated or foreign molecular patterns, PRRs initiate signaling pathways associated with activation of the innate immune response [37]. Three such PRRs that have been reported to be regulated by PACT are PKR, retinoic acid-induced gene (RIG)-I (as known as DDX58), and melanoma differentiation-associated protein (MDA)-5 (as known as IFIH1). RIG-I is activated by short double stranded (ds) RNAs, and 5′ tri- and di-phosphorylated dsRNAs that lack ribose 2’-O-methylation [40]. Once bound to a non-self RNA molecule, RIG-I initiates downstream signaling by triggering oligomerization of its caspase activation and recruitment domains (CARDs). Once oligomerized, RIG-I’s CARD domains in turn oligomerize with CARD domains in Mitochondrial Anti-Viral Signaling Protein (MAVS) (also known as IPS-1, VISA, and Cardif) and activate downstream signaling [38,41]. After MAVS activation, the transcription factors IRF-3 and NF-κB are activated, which induce type I interferon (IFN) genes [38,42]. The induced IFNs then initiate anti-viral signaling responses in both an autocrine and paracrine manner [43,44] by inducing additional gene products [45]. Some of the studies have reported PACT to directly interact with RIG-I to promote the oligomerization of CARD domains, ATPase activity, downstream MAVS activation, and IRF3 phosphorylation leading to enhanced IFN synthesis [35]. SiRNA-mediated knockdown of PACT was shown to inhibit RIG-I dependent IFN production during Sendai virus infection [35]. A direct interaction of PACT with LGP2 also is reported to regulate RIG-I- and MDA5-dependent IFN induction and PACT-LGP2 interaction is reported to be required for MDA5-mediated IFN production during Cardiovirus infection [46]. In addition, PACT is also reported to be an essential cofactor for dsRNA-induced formation of MDA5 oligomers [47].

Given the demonstrated regulatory role of PACT in dsRNA-induced IFN production, we investigated the effect of the most prevalent DYT-*PRKRA* mutation P222L on RIG-I-mediated IFN induction in response to dsRNA. Although the P222L mutation is recessive and most of the DYT16 patients are homozygous [3], one patient that developed acute onset dystonia during early childhood after a febrile illness carries a P222L mutation inherited from one unaffected heterozygous parent and another de novo C213R mutation in trans [10]. We, thus, examined both the homozygous P222L as well as P222L/C213R compound heterozygous DYT16 cells in this study. Our results indicate a stimulatory effect of the P222L mutation on the RIG-I pathway and suggest a possible involvement of type I IFNs in DYT-*PRKRA* pathophysiology to warrant further investigation of heightened type I IFN production and possible interferonopathy in DYT-*PRKRA* patients.

## 2. Materials and Methods

### 2.1. Reagents and Cell Lines

HEK293T (ATCC CRL-3216) cells were cultured in Dulbecco’s modified Eagle’s medium (DMEM) containing 10% fetal bovine serum and penicillin/streptomycin. The WT and DYT-*PRKRA* dystonia patient lymphoblast cell lines [22,24] were a kind gift from Dr. Nutan Sharma and were established from lymphocytes isolated from blood samples by Epstein–Barr Virus transformation to create stable cell lines as previously described [48]. The lymphoblast lines were cultured in RPMI 1640 medium containing 10% FBS and penicillin/streptomycin. Low molecular weight (LMW) Polyinosinic-polycytidylic (PolyI:C, 0.2–1 kb) was purchased from Invivogen (San Diego, CA, USA). The expression plasmid flag-RIG-I/pEFbos+ [49] was purchased from Addgene (plasmid #52877). IFN-β–luc [50] was a kind gift from Dr. Richard Randall and PACT and P222L expression plasmids were as described before [22]. The antibodies used were as follows: PACT: anti-PACT monoclonal (Abcam, ab75749); FLAG-HRP: anti-FLAG monoclonal M2-HRP (Sigma-Aldrich A8592, St. Louis, MO, USA); MYC-HRP: anti-MYC monoclonal (Santa Cruz, 9E10); β-Actin: Anti-β-Actin-HRP monoclonal (Sigma-Aldrich, A3854).

#### 2.1.1. Transfections and Luciferase Assays

Transfections of reporters, protein expression constructs, and polyI:C were completed using Effectene (Qiagen, Hilden, Germany) transfection reagent. dsRNA treatment was for 16 h overnight, starting at 24 h after reporter and protein vectors were transfected. Luciferase activity was determined using the Dual-Luciferase Reporter Assay System from Promega (Madison, WI, USA). Luciferase readings were normalized to Renilla expressed from pRL null. For all luciferase experiments, 200 ng of IFN-β–luc [50], 1 ng of pRL-null, 50 ng of RIG-I/pEFbos+ 50 ng of PACT/pcDNA3.1-, and 50 ng of P222L/pcDNA3.1- were transfected into HEK293Ts. HEK293T cells were harvested 16 h after dsRNA treatment (50 ng of polyI:C). Each sample was washed twice with 1X PBS then lysed in 200 µL of 1X Passive Lysis Buffer (Promega) for 5 min. Lysates were spun at 13.2 k for 5 min and 15 µL of each lysate was transferred to a new tube. Then, 25 µL of both Luciferase and Stop and Glo-Renilla reagents (Promega) were used for readings. Enzymatic activity was measured using a luminometer. Each test was completed in triplicate and repeated twice. For each test, firefly luciferase numbers were first normalized to Renilla luciferase numbers to normalize for transfection efficiency. Then, control numbers were set as 1 relative luciferase unit (RLU) and all samples within that set were compared to that. 

#### 2.1.2. Western Blot Analysis

Lymphoblasts derived from DYT16 patients either homozygous for P222L or compound heterozygous containing P222L and C213R mutations as independent alleles were cultured alongside lymphoblasts derived from unaffected (wt) persons containing no mutations in PACT. Cells were harvested in RIPA (150 mM NaCl, 1.0% IGEPAL^®^ CA-630, 0.5% sodium deoxycholate, 0.1% SDS, 50 mM Tris, pH 8.0) buffer containing a 1:100 dilution of protease inhibitor cocktail (Sigma) and phosphatase inhibitor (Sigma). Concentration of total protein extract was then determined using BCA assay and appropriate amounts of extracts were analyzed by Western blot analyses using appropriate antibodies as indicated in the figure legend. In the case of Western blot analysis of transfected cells, HEK 293 cells were harvested in Passive Lysis Buffer (Promega) for determining luciferase activities in the samples. After this, the total protein concentrations in the extracts were determined using a BCA assay and appropriate amounts of extracts were analyzed by Western blot analyses using appropriate antibodies as indicated in the figure legend.

#### 2.1.3. RNA Isolation and qRT-PCR

Total RNA was isolated from lymphoblasts using RNAzol RT (Sigma-Aldrich). After two washes with ice-cold PBS, 250 µL of RNAZol RT was added and total RNA was isolated as per the manufacturer’s instructions. For each sample, we reverse transcribed 800 ng of RNA using kit iScript™ Reverse Transcription Supermix for RT-qPCR (Bio-Rad, Hercules, CA, USA). The expression analysis of IFN β and five ISGs was performed using the IFNr qRT-Primers from Invivogen (Catalog # rts-hifnr) and GAPDH primers for quantification as a control housekeeping gene. TaqMan Universal PCR Master Mix (Applied Biosystems, Waltham, MA, USA) and cDNA derived from 40 ng total RNA was used. All reactions were run on a BioRad CFX96 Real-Time System C1000 thermal cycler machine using the conditions recommended for the primer sets by Invivogen. For each patient, relative quantification (RQ) (2−ΔΔCt) [38], i.e., the normalized fold change relative to the mean of each ISGs of the controls, was calculated.

#### 2.1.4. IFN β Induction in Response to dsRNA in Lymphoblasts

Transfection of reporters, protein expression constructs, and polyI:C was completed using Effectene (Qiagen) transfection reagent. dsRNA treatment was for 16 h overnight, starting at 24 h after reporter vectors were transfected. Luciferase activity was determined using the Dual-Luciferase Reporter Assay System from Promega. Luciferase readings were normalized to Renilla expressed from pRL null. Then, 200 ng of IFN-β–luc [50], 1 ng of pRL-null, and 250 ng of empty vector pcDNA3.1- (as filler DNA to achieve transfection efficiency) were transfected into the lymphoblasts at 100,000 cells/mL. A total of 24 h after transfection, LMW polyI:polyC complexed with transfection reagent LyoVec™ (Invivogen, Cat # tlrlpicwiv) was added to the culture medium at 100 ng/mL final concentration and the cells were harvested 16 h after dsRNA treatment. The LyoVec complexed dsRNA efficiently enters mammalian cells to induce RIG-I signaling. Each cell pellet was washed twice with 1X PBS then lysed in 200 µL of 1X Passive Lysis Buffer (Promega) for 5 min. Lysates were spun at 13.2 k for 5 min and 15 µL of each lysate was transferred to a new tube. In total, 25 µL of both Luciferase and Stop and Glo-Renilla reagents (Promega) were used for reading the enzymatic activity using a luminometer. Each test was completed in triplicate and repeated 2 times. For each test, firefly luciferase numbers were first normalized to Renilla to normalize for transfection efficiency. Then, control numbers were set as 1 relative luciferase unit (RLU) and all samples within that set were compared to that. 

#### 2.1.5. Statistics

To determine statistical significance when analyzing the various assays performed in this study, we executed a two-tailed Student’s T-test, assuming equal variance. Each figure denotes significant *p* values; note that our alpha level was 0.001.

## 3. Results

**Figure 1:**
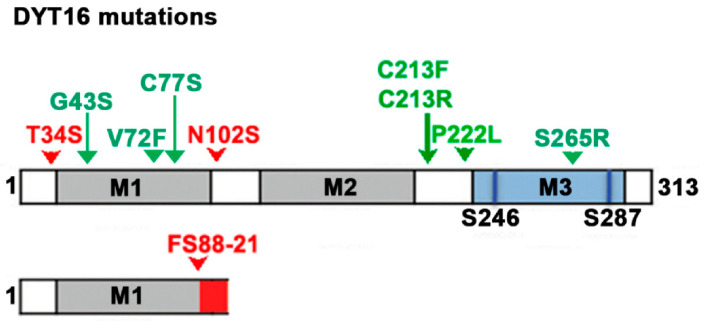
Schematic representation of DYT-*PRKRA* mutations: of the three conserved dsRBMs, M1 and M2 are shown in grey and the third motif lacking dsRNA-binding (M3) is shaded blue with the two phosphorylation sites represented as dark blue lines. Dominant DYT-*PRKRA* mutations are indicated in red while recessive mutations are indicated in green. The dominant frameshift mutation truncates the protein after the first 88 amino acids with 21 extraneous amino acids (red) shown at the carboxy terminus.

The schematic in Figure 1 depicts the locations of the reported DYT-*PRKRA* mutations relative to the conserved dsRNA-binding domains (M1 and M2) and PKR activation domain (M3) [13,51]. The two mutations in this study, P222L and C213R, reside outside the known functional domains of PACT important for its interaction with PKR, TRBP, or with dsRNA. As we have previously reported, both these mutations have no effect on PACT’s dsRNA-binding but P222L mutant enhances PACT’s interaction with PKR and both P222L and C213R mutations enhance PACT–PACT interactions [22,24]. In addition to regulating PKR activity, PACT also affects the IFN production in response to dsRNA [52]. Thus, we examined the effect of the most prevalent DYT-*PRKRA* substitution mutation P222L on PACT’s ability to enhance IFN production. In addition, we examined IFN production in DYT-*PRKRA* patient lymphoblasts from P222L homozygotes and compound heterozygote carrying P222L and C213R mutations. The P222L mutation is recessive and all reported DYT16 cases are homozygous except for one patient, who developed acute onset dystonia in early childhood after a febrile illness and was later confirmed by exome sequencing to carry an inherited P222L mutation from one unaffected parent and a de novo C213R mutation in trans [10].

### 3.1. PACT Enhances RIG-I-Mediated IFN Induction and P222L Enhances It Further

**Figure 2:**
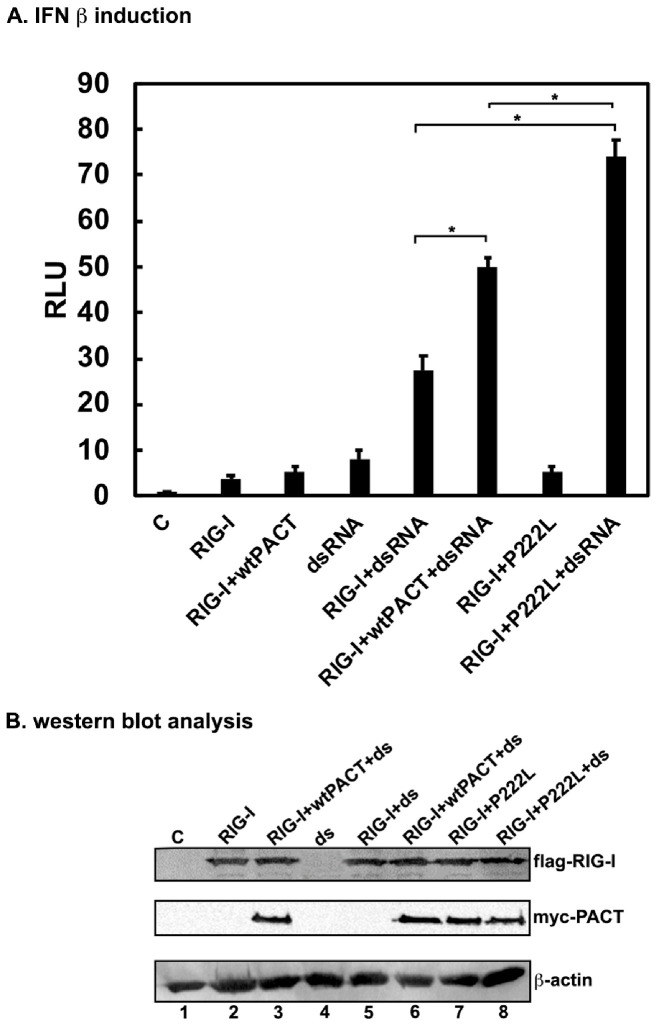
P222L mutation augments PACT and RIG-I-mediated IFN β production in response to low molecular weight (LMW) polyI:C. (**A**) HEK293Ts were transfected with IFN-β-Luc reporter alone (C and ds), or IFN-β-Luc reporter and expression construct for flag-RIG-I, and either 50 ng of myc-tagged wt PACT or P222L mutant expression construct as indicated, then treated with polyI:C 24 h after transfection as indicated (dsRNA). pRLnull plasmid was co-transfected in all samples for normalization of transfections. Cell extracts were assayed for dual luciferase activity 16 h after polyI:C treatment. *: The *p*-values based on 3 independent experiments that were <0.001 and are denoted as significant with an asterisk. (**B**) Western blot analysis of transfected cells. Whole cell extracts prepared at 24 h from transfected cells were analyzed by Western blot analysis. The samples 1–8 correspond to the order of samples in part A. Blots were probed with anti-flag, anti-myc, and anti-β-actin antibodies for flag-RIG-I, myc-PACT, and endogenous β-actin used for ensuring equal loading in all lanes.

It has been reported that PACT plays a stimulatory role in IFN β induction in response to dsRNA by interacting directly with RIG-I and enhancing its ATPase activity [35] and also by stimulating the MDA5 pathway [46,47]. Thus, we tested the effect of P222L mutation on PACT’s ability to enhance IFN β induction via RIG-I. HEK293 cells were co-transfected with various combinations of RIG-I and PACT expression constructs, IFN β-luciferase reporter and pRLnull for normalization of transfection efficiencies as indicated. Then, 24 h after transfection, the cells were treated with LMW (0.2–1 kb) poly(I:C) and 16 h later luciferase activities were measured. As seen in Figure 2, wt PACT enhanced RIG- mediated IFN β induction in response to dsRNA by about 1.8-fold, and P222L mutant enhances it by about 2.7-fold, indicating that the P222L mutation augments PACT’s RIG-I stimulatory activity. The effect of P222L mutation on PACT’s ability to enhance IFN induction in response to dsRNA seems moderate but highly reproducible, as we saw similar effects in multiple replicates of this experiment. To ensure that P222L expression levels were comparable to wt PACT, we performed Western blot analyses with anti-myc antibody as the PACT expressed from the transfected plasmids is tagged with a myc tag. The expression levels of RIG-I expressed from the transfected expression construct were also assessed using an anti-flag antibody as the RIG-I carried a flag tag. As seen in Figure 2B, the expression of P222L mutant was similar to wt PACT, thereby confirming that the enhanced expression of IFN β was a result of the mutation itself and not due to differences in expression levels of PACT. The expression level of RIG-I was also consistent in all samples of the transfection experiment, supporting our conclusions.

### 3.2. Basal Levels of IFN β and ISGs Are Higher in Lymphoblasts Established from DYT-PRKRA Patients

**Figure 3:**
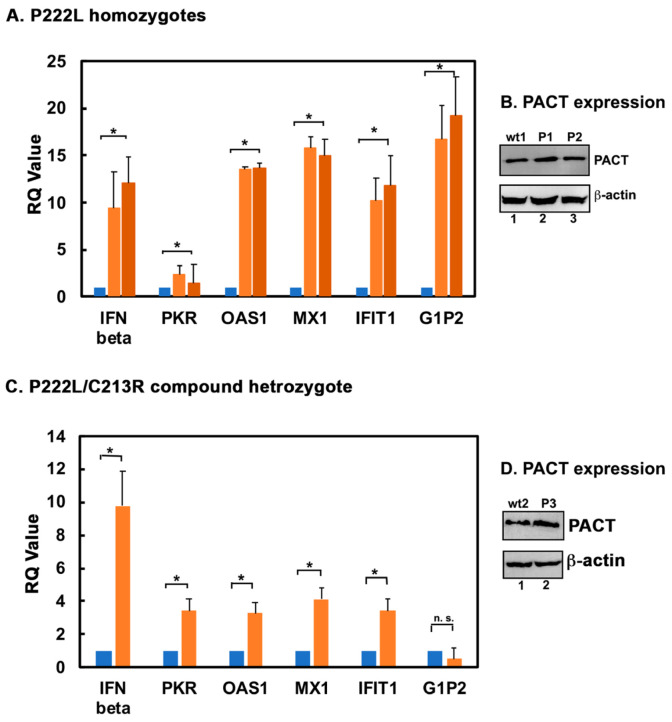
DYT-*PRKRA* patient lymphoblasts express higher basal levels of IFN β and ISG mRNAs. Quantitative RT-PCR of a panel of IFN β and 5 ISGs in lymphoblasts established from DYT-*PRKRA* patients (orange bars) and unaffected family members (blue bars). The RQ values indicate that IFN β and ISG expression was upregulated (* *p* < 0.001, indicated by asterisk) in DYT-*PRKRA* cells compared to unaffected control cells. (**A**) qRT-PCR analysis of two P222L homozygous patients and one unaffected individual. IFN β and all ISGs were upregulated in DYT-*PRKRA* patient samples. (**B**) Western blot analysis. Whole cell extracts prepared from wt (wt1) and patient (P1 and P2) lymphoblasts were analyzed by Western blot analysis. Blots were probed with anti-PACT, and anti-β-actin antibodies with β-actin used for ensuring equal loading in all samples. (**C**) qRT-PCR analysis of one compound heterozygous DYT-*PRKRA* patient and one unaffected family member. IFN β and four of the five ISGs were upregulated in the DYT-*PRKRA* patient sample, n. s. denotes that the difference in expression of G1P2 was not significant. (**D**) Western blot analysis. Whole cell extracts prepared from wt (wt2) and patient (P3) lymphoblasts were analyzed by Western blot analysis. Blots were probed with anti-PACT, and anti-β-actin with β-actin was used for ensuring equal loading in all samples.

We examined the basal expression levels of IFN β and a few ISGs by qRTPCR in DYT-*PRKRA* patient lymphoblasts from two different genotypes in comparison with normal (wt) lymphoblasts. We had two normal (wt) samples and two DYT-*PRKRA* samples homozygous for the P222L mutation [22]. The two wt samples showed nearly identical levels of IFN β and ISG mRNAs; thus, only one is represented in Figure 3A. The P222L homozygous cells had between 9- and 15-fold elevated expression of IFN β and ISGs, respectively, as compared to the wt cells. To investigate if the expression levels of PACT protein were different in wt and patient lymphoblasts, we used Western blot analysis. As seen in Figure 3B, the expression level of P222L mutant PACT protein is the same as the wt PACT protein confirming that the differences in IFN β and ISG expression arise from the mutation and not from changed expression level of the P222L protein. In addition, we also used one normal (wt) and one DYT-*PRKRA* patient’s lymphoblasts that were compound heterozygous with P222L and C213R mutations (Figure 3C) [24]. The data demonstrates that, similar to P222L homozygous lymphoblasts, the P222L/C213R heterozygous lymphoblasts also show between 3.5- and 9.7-fold elevated expression of IFN β and four of the five ISGs, respectively. The expression level of PACT protein was comparable in the wt and patient lymphoblasts as seen in Figure 3D, confirming that the increase in IFN β and ISG expression is a result of PACT mutations. 

### 3.3. DYT-PRKRA Patient Lymphoblasts Produce Higher Levels of IFN β after Induction with dsRNA

**Figure 4:**
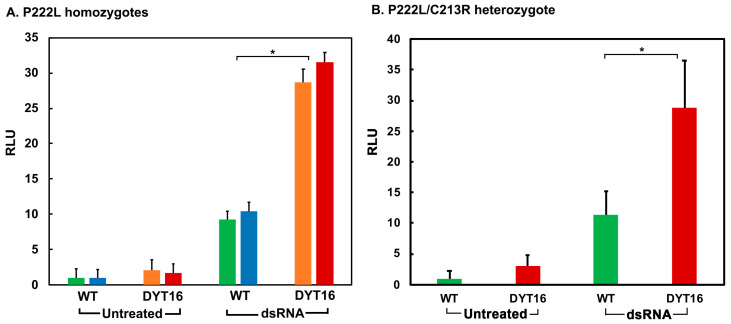
Enhanced induction of IFN β in DYT-*PRKRA* patient lymphoblasts in response to dsRNA. DYT-*PRKRA* and normal lymphoblasts were transfected with IFN-β-Luc reporter and pRLnull plasmids, then transfected with LMW polyI:C at 24 h after the plasmid transfections. Cell extracts were assayed for dual luciferase activity 16 h after polyI:C treatment. (**A**) IFN β induction in P222L homozygous lymphoblasts. Green and blue bars: lymphoblasts from two unaffected (wt) individuals. Orange and red bars: lymphoblasts from two unrelated DYT-*PRKRA* individuals. *: The *p*-values (indicated by asterisk) based on 3 independent experiments with each sample in triplicates were significant and <0.001. (**B**) IFN β induction in P222L/C213R compound heterozygous lymphoblasts. Green bars: lymphoblasts from one unaffected family member. Red bars: lymphoblasts from an DYT-*PRKRA* individual. The *p*-values (indicated by asterisk) based on 3 independent experiments with each sample in triplicates were significant and <0.001.

In order to test if IFN β is induced at higher levels in DYT-*PRKRA* cells in response to dsRNA, we utilized both the DYT-*PRKRA* patients’ lymphoblasts homozygous for the P222L mutation [22] as well as the ones from compound heterozygote carrying P222L and C213R mutations [24]. Similar to Figure 3, for homozygous patients, we compared lymphoblasts from two normal (wt) family members of the respective probands and two unrelated patients (probands). For the compound heterozygote patient, we used the lymphoblasts from a single patient sample and one related, unaffected wt family member. We co-transfected IFN β-luciferase reporter with pRLnull (for normalization of transfection efficiencies) in the lymphoblasts and stimulated them with low molecular weight (LMW, 0.2–1 kb) poly(I:C) 24 h after the transfection. Then, 16 h after the dsRNA stimulation the luciferase activity was assayed. As seen in Figure 4A, the both patient lymphoblast cell lines of P222L homozygous genotype showed slightly elevated (about 1.2- and 2-fold higher than wt) IFN β-luciferase activity at basal levels. After dsRNA induction, the wt cells showed about 10-fold induction of IFN β promoter and the DYT-*PRKRA* lymphoblasts showed about 30-fold higher IFN β promoter activation; thus, indicating that when stimulated by dsRNA, the patient cells produce higher levels of IFN β. In the case of the compound heterozygote, the patient lymphoblast cell line showed slightly elevated (about 2-fold higher than wt) IFN β-luciferase activity at basal levels. After dsRNA induction the wt lymphoblasts showed about 11-fold induction of IFN β promoter and the patient lymphoblasts showed about 29-fold higher IFN β promoter activation. These results indicate that the established DYT-*PRKRA* lymphoblast lines show about 3-fold higher IFN β induction in response to dsRNA both for homozygous P222L as well as for P222L and C213R compound heterozygous patients.

We also investigated the induction of ISGs in response to dsRNA in the DYT-*PRKRA* patient lymphoblasts in comparison with the wt lymphoblasts. Unlike IFN β, we observed only slightly enhanced induction of ISGs in response to dsRNA and the response of wt and DYT-*PRKRA* cells to dsRNA was comparable. As PACT has been reported to interact directly with RIG-I and enhance its dsRNA-dependent ATPase activity [35], we examined if the P222L mutation causes enhanced interaction with RIG-I using co-immunoprecipitation (coIP) assays in HEK293 cells transfected with RIG-I and PACT expression plasmids (data not shown). However, contrary to the published report [35], we detected no direct interaction between RIG-I and wt PACT or P222L mutant. The reason for this discrepancy is unknown at present, but it is possible that in lymphoblasts, the stimulatory effect of PACT on IFN production may work without a direct interaction with RIG-I. Further studies are essential to characterize PACT’s stimulatory effect on RIG-I-mediated IFN induction in response to dsRNA and how DYT-*PRKRA* mutations may affect this pathway.

## 4. Discussion

Dystonia is a clinically heterogenous group of movement disorders characterized by involuntary muscle contractions and painful twisting movements that can often result in abnormal postures [53]. The etiology of dystonia is broad and several inherited monogenic types have been identified in recent years [54]. In order to develop effective therapeutic options, it is essential to understand the affected molecular pathways. In the case of DYT1, cellular localization studies of mutant torsinA protein led to a cell-based screening for compounds that could correct its mislocalization and resulted in the identification of Ritonavir as a possible therapeutic option [55].

With the goal of defining the effect of pathological mutations in *PRKRA*-encoded PACT protein on various cellular pathways, we initially characterized the effects of one dominant and six recessive mutations on the ISR pathway in response to endoplasmic reticulum (ER) stress [22,24]. In the current study, we examined how the most prevalent DYT-*PRKRA* mutation P222L affects type I IFN production. PACT is essential for IFN production in response to viral infection [35,36,46,47] and many viruses target PACT inactivation in order to replicate efficiently [34,56,57,58,59,60,61,62]. This naturally raises a question if in addition to causing maladaptive ISR signaling, DYT-*PRKRA* mutations also may exert an effect on the innate immune system by altering type I IFN production. Our results indicate that P222L mutation causes enhanced IFN β production in response to dsRNA via the RIG-I-induced signaling. More importantly, the basal levels of IFNβ as well as ISG mRNAs were elevated in DYT-*PRKRA* patient lymphoblasts as compared to control lymphoblasts. These results indicate a possible involvement of type I IFNs in DYT-*PRKRA* pathophysiology, an aspect that needs to be investigated in depth in future. Although our study can be considered groundbreaking, certain limitations of our study need to be emphasized. We studied only one DYT-*PRKRA* mutation and it is unknown if enhanced IFN production is a common feature of all other mutations reported thus far. Additionally, we examined IFN and ISG expression using established DYT-*PRKRA* lymphoblast lines and not blood samples from DYT-*PRKRA* patients, which may have given higher fold differences in IFNβ and ISG expression levels compared to unaffected controls. Such blood samples were currently unavailable to us and need to be included in future analyses. It may also be useful to establish cell lines carrying specific DYT-*PRKRA* mutations using gene editing approaches. As the DYT-*PRKRA* patient cells exhibit maladaptive ER stress response and altered eIF2α signaling, which is a characteristic of more than one form of inherited dystonia, the extent of to which enhanced production or signaling of type I IFNs contributes to DYT-*PRKRA* pathophysiology requires further investigation. 

In support of the DYT-*PRKRA* dystonia symptoms possibly being a result of IFN overproduction, it has been documented that people undergoing type I IFN therapy for hepatitis B and C develop dystonia as a side effect [63,64]. Neurologic symptoms of IFN therapy, such as psychomotor slowing, fatigue, parkinsonism, and dystonia during IFN treatment are thought to involve alterations in basal ganglia circuitry [65,66]. Cancer patients undergoing type I IFN therapy also are reported to show tremor and rigidity indicating that IFN therapy may have anti-dopaminergic effects [67]. Type I IFN administration in mice inhibits dopaminergic activity and induces motor symptoms [68]. Multiple sclerosis patients on IFN therapy are also reported to develop dystonia and gait disturbances as a side effect [69]. Our results agree with the reports of dystonia observed as a side effect in patients undergoing IFN treatment for various pathological conditions.

Dystonia has been noted as one of the symptoms of the pathological conditions classified as “interferonopathies” that result from overproduction of type I IFNs or enhanced IFN signaling [70,71,72]. The first report of a Mendelian disease arising from enhanced type I IFN production and signaling was of Aicardi–Goutieres syndrome (AGS) that described increased IFNα levels and activity in serum and cerebrospinal fluid (CSF) of the affected children [73]. The symptoms of this disorder resembled those of viral infections acquired in utero, which also suggested IFN involvement [74,75]. Recombinant type I IFN, used for long term therapy in cancer patients, was reported to give rise to termed systemic lupus erythematous (SLE), an autoimmune disease [76,77]. A recent review of interferonopathies describes 38 different Mendelian genotypes that encompass involvement of nucleic acid metabolism or sensing pathways, type I IFN receptor signaling, maintenance of mitochondrial integrity, and proteasome activity [78]. A significant percentage of patients with interferonopathies exhibit dystonia as one of the symptoms among numerous other neurological and inflammatory phenotypes [79,80]. Inflammatory problems have not been described in DYT-*PRKRA* patients, and it is unclear at present if the relatively milder upregulation of IFN production or signaling could possibly result only in dystonia without other associated inflammatory symptoms. The upregulation of IFN β production and ISGs we observed in DYT-*PRKRA* cells is lower compared than that found in the interferonopathies [73,81,82,83], which may be due to our analysis being performed with established lymphoblast lines instead of patient blood and serum samples. Alternately, as PACT is generally considered to be an accessory factor that augments the RIG-I signaling pathway and without PACT RIG-I can still trigger IFN production (Figure 2, [84]), the effect of DYT-*PRKRA* mutation P222L can only be expected to be modest at its best. It is possible that the moderately high expression of IFN β and ISGs could possibly contribute to the dystonia symptoms in these patients without causing more severe inflammatory phenotypes observed in AGS, Singleton–Merten syndrome (SMS), and SLE. In our transfection experiments using HEK293 cells (Figure 2), the effect of P222L mutation also seems modest but is consistently reproducible. The presence of endogenous wt PACT in HEK293 cells could reduce the stimulatory effect of P222L mutation, as P222L is recessive and heterozygous individuals do not show a dystonia phenotype [3].

Prolonged or enhanced PKR activation by DYT-*PRKRA* mutations in PACT results in increased apoptosis in patient cells in response to ER stress [22,24]. Although neurodegeneration is the expected long-term outcome of enhanced apoptosis, a systematic search for neurodegeneration has so far not been investigated in DYT-*PRKRA* patients. Thus far, over 30 DYT-*PRKRA* patients with *PRKRA* mutations have been identified. Brain magnetic resonance imaging (MRI) revealed progressive volume loss of the bilateral basal ganglia initially in one compound heterozygous patient with P222L and C213R mutations [10] and bilateral striatal degeneration (BSD) in two additional compound heterozygous patients, one carrying point mutations P222L and G34S and the other carrying mutations C213F and V72F [11]. The most direct evidence for involvement of hyperactive PKR in dystonia comes from Kuipers et al. [85], who identified hyperactive PKR variants and enhanced eIF2α phosphorylation in early-onset dystonia. Onset of dystonia or neuronal deterioration in the context of febrile illness or general anesthesia was noted in some patients. De novo missense variants in PKR also have been reported to cause a complex neurodevelopmental condition that resembles the vanishing white matter disease [86]. Of the eight patients, dystonia was present in five and all eight patients exhibited neurological regression in the setting of a febrile illness. An additional PKR mutation was recently reported with brain MRI abnormalities and childhood-onset episodes of neurological decompensation and dystonia after a febrile illness [87]. As PKR activation occurs during virus infections and physiological stress, it is perceivable that onset of dystonia following a febrile illness arose due to hyperactive PKR. All these observations suggest a role of the PKR hyperactivity in a group of conditions characterized by BSD and deterioration episodes with dystonia following stressors such as a febrile illness. 

PKR activity regulated by dsRNA or PACT has been established to play a central role in regulation of inflammation and innate immunity [19]. As a cytoplasmic dsRNA sensor, PKR is known for its essential function in induction of type I IFNs during viral infections but the exact mechanism varies depending on the virus [88,89,90]. In addition, PKR activation by PACT occurs in response to several stimuli such as oxidative stress, hyperosmolarity, serum deprivation, and ER stress that potentially can lead to various inflammatory conditions [19]. In our current study, we detected elevated expression of PKR in the compound heterozygous DYT-*PRKRA* lymphoblasts (Figure 3B). Previously, we reported higher PKR expression, and increased levels of phosphorylated, active PKR, as well as higher basal level of apoptosis in these lymphoblasts [24]. Additionally, the contribution of PKR in idiopathic inclusion body myositis (IBM), which is an age-related autoimmune muscular pathology with a type I IFN signature, has recently been proposed [91,92,93]. Although we did not examine the contribution of enhanced PKR activity in DYT-*PRKRA* cells to IFN production in the current study, it is certainly possible that hyperactive PKR exerts additional regulation for type I IFN induction. In summary, these studies present a new paradigm for future mechanistic investigations on DYT-*PRKRA* that may uncover novel drug targets and potentially lead to effective therapies for DYT-*PRKRA*.

## Data Availability

Data sharing is not applicable to the paper, because all experimental procedures and data encompassing this work are included in this manuscript.
